# A clinical study of liposuction followed by lymphovenous anastomosis for treatment of breast cancer-related lymphedema

**DOI:** 10.3389/fsurg.2023.1065733

**Published:** 2023-03-15

**Authors:** Kun Chang, Song Xia, Chen Liang, Yuguang Sun, Jianfeng Xin, Wenbin Shen

**Affiliations:** Department of Lymph Surgery, Capital Medical University Affiliated Beijing Shijitan Hospital, Beijing, China

**Keywords:** lymphedema, treatment, liposuction, upper limb, lymphovenous anastomosis, breast cancer-related lymphedema

## Abstract

**Objective:**

In this work, we studied the clinical effect of liposuction followed by lymphovenous anastomosis (LVAs) for the treatment of breast cancer-related lymphedema (BCRL).

**Methods:**

We analyzed 158 patients with unilateral upper limb BCRL who underwent liposuction followed by LVAs 2–4 months later. Arm circumferences before and 7 days after the combined treatments were prospectively recorded. Circumferences of different upper extremities were measured before the procedure, 7 days after LVAs, and during the follow-ups. Volumes were calculated with the frustum method. During the follow-ups, the conditions of patients’ treated arms, i.e., the frequency of erysipelas episodes and dependence on compression garments, were recorded.

**Results:**

The mean circumference difference between two upper limbs decreased significantly from M (P25, P75) of 5.3 (4.1, 6.9) preoperatively to 0.5 (−0.8, 1.0) (*P* < 0.05) 7 days after treatments, while at follow-up 0.3 (−0.4, 1.0). The mean volume difference decreased significantly from M (P25, P75) of 838.3 (662.4, 1,129.0) preoperatively to 7.8 (−120.3, 151.4) (*P* < 0.05) 7 days after treatments, while at follow-up 43.7 (−59.4, 161.1). The incidence of erysipelas also significantly decreased (*P* < 0.05). 6.3% of patients were already independent of compression garments during the past six months or even more.

**Conclusion:**

Liposuction followed by LVAs is an effective method for the treatment of BCRL.

## Introduction

Lymphedema is a chronic condition that is usually not life-threatening but can cause quite a deleterious impact on the quality of life. The swelling limb also reduces functionality and increases the risk of comorbidities, anxiety, and depression (1, 2). Insufficiency or obstruction of lymph backflow caused by lymphatic vascular malformations or secondary factors such as trauma, infection, or mostly oncologic therapy leads to fluid accumulation of extremities. The condition of excess infiltration of interstitial fluid rich of protein then causes fibrosis and sclerosis of skin and adipose tissues, which finally ends up with the increase of fluid and tissue volume of limbs (3–6). Breast cancer-related lymphedema (BCRL) is currently the leading cause of secondary lymphedema in the upper limbs. Lymphedema in BCRL usually happens at the ipsilateral upper limb. It is reported that the incidence of BCRL in breast cancer patients is 3%–65% depending on the factors such as surgical options, wound healing of the surgical area, radiation therapy, invasive manipulation of the ipsilateral limb, body mass index (BMI), delayed recovery of the affected limb function, and repeated infection of the affected limb (7–12). The survival rate of breast cancer has been continuously improved with the development of the diagnosis and treatment. As a consequence, more and more attention is paid to the complications, for instance, BCRL. In addition, the risk of erysipelas occurring on the lymphedema limb is especially higher than normal occasions, which makes BCRL one of the complications that impairs the long-term life quality most seriously.

Lymphatic obstruction leads to the increase of lymphatic hydrostatic pressure, which causes progressive damage of lymphatic structures manifesting as dilated lymphatic vessel, impaired valve function, and distal lymph stasis. Long-term stasis of lymph rich of nutrient leads to tissue deposition of fat, fibrosis, repeated erysipelas, and thickening of extremities eventually. It is a vicious cycle that the increase of cutaneous and subcutaneous lesions produces more lymph, which in turn increases lymph pressure (13, 14).

The treatment of BCRL includes nonsurgical and surgical treatments. The International Society of Lymphology reported four clinical stages of lymphedema. The latent or subclinical stage, defined as stage 0, refers to the impaired lymph transport without extremity lymphedema. Stage I represents an early fluid accumulation presented as pitting edema that subsides with limb elevation. Stage II signifies pitting and fibrosis, which manifests that limb elevation alone rarely reduces tissue swelling. Stage III encompasses lymphostatic elephantiasis where pitting is absent and trophic skin changes, such as acanthosis, fat deposits, and warty overgrowths. The severity within each stage is based on the volume differences between two upper limbs: minimal (<20% increase), moderate (20%–40% increase), and severe (>40% increase). Patients with BCRL at the early stage of lymphedema mostly get relieved with conservative therapies (15). Those refractory to conservative treatments and are more severe will receive surgical treatments. At present, the most common surgical methods for limb lymphedema are debulking procedures (including excisional surgery and liposuction) and lymphatic reconstructions (including lymphovenous anastomosis (LVAs), vascularized lymph node transfer/transplantation (VLNT), and lymphatic grafting) (16, 17). When the excess volume is dominated by dermato-lipo-fibrosclerosis tissue instead of accumulated lymph, conservative treatment is limited. Liposuction enables the removal of the adipose tissue besides accumulated lymph, which microsurgical reconstructions cannot achieve. Compared with the traditional excisional surgery, liposuction is safer and lesser invasive and can be perform twice (18, 19). While liposuction can effectively reduce limb volume and is aimed at the process from hyperplasia to lymph stasis in the pathophysiological cycle of lymphedema, the lymph flow is still obstructed without effective lymphatic flow pathways. As a result, lymphedema recurs and aggravates gradually with the accumulation of lymph fluid. When the pressure in the lymphatic vessel is higher than in the vein, lymph fluid smoothly flows from lymphatic vessels to the vein. In this aspect, lymphatic reconstructions can effectively improve the lymphatic flow and reduce the lymphatic stasis of the affected limb.

For the first time, the combination of liposuction and LVAs in the treatment of BCRL was carried out widely in the Department of Lymphatic Surgery, Beijing Shijitan Hospital, and the outcome was quite satisfactory in this study.

## Patients and methods

This study was approved by the Medical Ethics Committee of Beijing Shijitan Hospital [sjtkyll-lx-2020(50)].

Patients with unilateral upper limb BCRL admitted to the Department of Lymphatic Surgery, Beijing Shijitan Hospital, from November 2015 to February 2018 were reviewed in this study. Patients’ demographics, breast cancer surgical approach, chemical and radiation therapy, operative notes, circumferences of arms, and episodes of erysipelas were prospectively recorded.

During the follow-ups, the patients’ conditions of both arms, including frequency of erysipelas episodes and dependence on compression garments, were recorded.

### Inclusion and exclusion criteria

All patients at the stages II and III of BCRL with the severity of moderate and severe within the forearm or upper arm received conservative treatments at the beginning. Patients refractory to conservative treatments and, thus, underwent liposuction followed by LVAs 2–4 months later were included.

Patients who were discovered to have recurrence of malignant tumor or comorbidities of venous reflux disorders as well as those who had edema of contralateral limb were excluded. Patients with symptomatic cardiovascular diseases or other diseases with contraindications to adrenaline injections were excluded. Patients who received other complementary therapies without authorization during the follow-up period were also excluded from this study.

### Measurement and calculation

The circumferences at the lower and upper third of the forearm and upper arm, elbow, and both upper extremities were measured by a particular therapist before the procedure of liposuction as the control for the response to therapy. Patients underwent liposuction followed by LVAs 2–4 months later. The circumferences at the same points were measured 7 days after the operation of LVAs on the day of discharge and during the follow-ups (23–36 months later) by the same therapist. The volumes of the extremities were calculated with the frustum method (20). Finally, the circumferential and volume difference between bilateral arms were calculated. The reduction rate from before the treatments to after treatments and during the follow-ups was compared.

### Surgical techniques

#### Liposuction

The patient was in the supine position under general anesthesia, and transverse skin incisions 3–5 mm in length were made on the ulnar side of the wrist, the ulnar and radial side of the forearm, and the radial side of the upper arm. To minimize blood loss, tumescence (containing 1 mg adrenaline, 0.2 g lidocaine, and 0.5 g sodium bicarbonate per 1 L of saline) was infiltrated according to the degree of the swelling of the affected limb. Next, Power-Assisted Pneumatic Liposuction Machine (PAPLM) with a negative atmospheric pressure of approximately 0.8–0.9 atm was used to perform liposuction with 15- to 25-cm-long cannulas. The cannulas should be as parallel as possible to the longitudinal axis of the upper limbs. After all the target subcutaneous adipose tissue and lymph fluid were absorbed, and subcutaneous drainage tubes were placed, sterilized compression (crushed gauze, cotton pad, and elastic bandage) is applied to stem the bleeding and reduce the postoperative edema. The drainage tubes and compression were removed 3 days later, and a compression garment was put on instead.

#### Lymphovenous anastomosis

**Figure 1:**
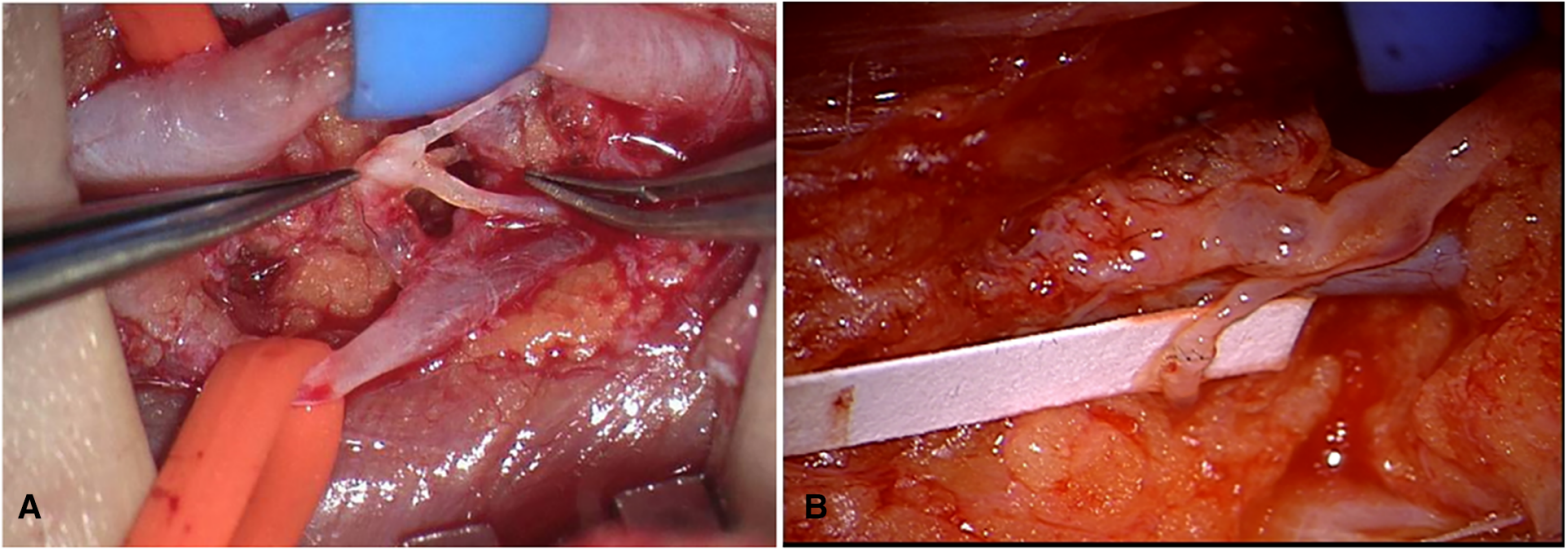
(**A**) The brachial artery was marked with blue tape and brachial vein tributary with orange tape; the lymphatics were anastomosed into brachial vein tributary. (**B**) The vein was flushed with lymph fluid and then turned white.

A 5 cm-long skin incision at the middle third of the upper arm was performed along the medial bicipital sulcus under general anesthesia. The brachial artery, vein, and the median nerve was exposed, among which the deep lymphatics were found using a microscope with magnification (12.5×4–6×). One or more brachial vein tributaries without reflux that matched the number and diameter of lymphatic were chosen. The vein tributary was cut leaving the distal end ligated, and the proximal part was flushed thoroughly with heparinized saline (6,250 U/500 ml) in case of any thrombus left. If there was reflux at the proximal end, the vessel wall will be narrowed circularly with the method of femoral vein valve repair. Then, all these lymphatic vessels were cut off and anastomosed into one (or more if necessary) venous lumen with 10-0 or 11-0 Prolene sutures, and the venous lumen would be narrowed in general (Figure 1). Complex decongestive therapy (CDT) was applied postoperatively, and a compression garment was replaced 1 month later.

### Statistical analysis

The statistical analysis was performed using SPSS 24.0 statistical software. The measurement data did not conform to the normal distribution after Shapiro–Wilk test, so it was represented by M (P25, P75). The differences among the data before and after treatment were assessed using the Friedman test and then the multiple comparisons were carried out with the Wilcoxon test.

## Results

### Patient characteristics

**Figure 2:**
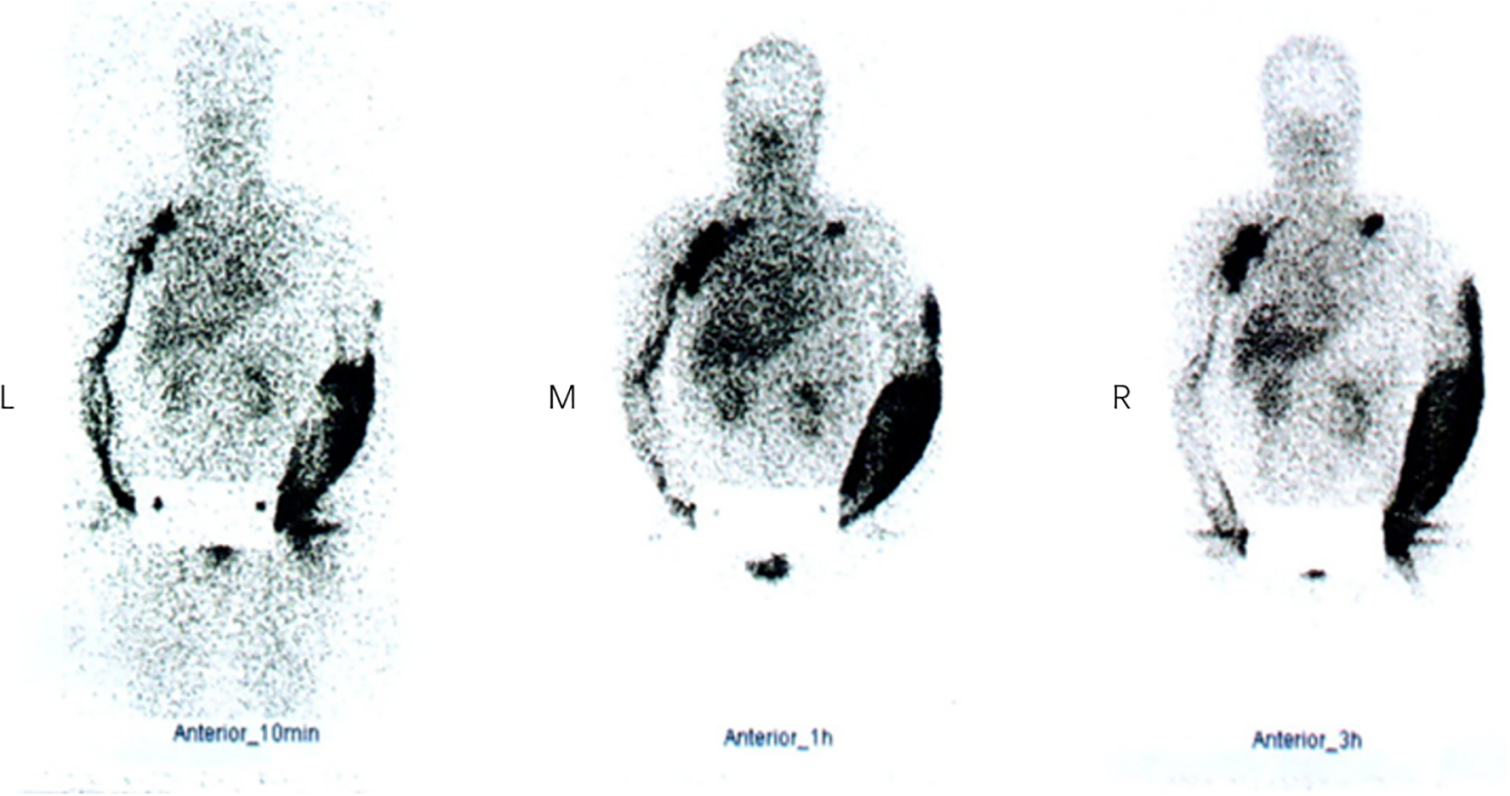
Lymphoscintigraphy in a BCRL patient at 10 min (left), 1 h (middle), and 3 h (right). Neither lymphatic vessels of left upper limb nor the left axillary lymph nodes were visualized. Imaging agents were distributed diffusely in the left arm subcutaneously, and a supraclavicular lymph node was visualized. Lymphatic vessels of right upper limb and the right axillary and subclavian lymph nodes were clearly visible and imaging agents flowed back smoothly. BCRL, breast cancer-related lymphedema.

From November 2015 to February 2018, a total of 179 patients with BCRL received treatments in our department. All the patients underwent the same preoperative evaluation, including lymphoscintigraphy for confirmed diagnosis (Figure 2) and Doppler ultrasound for the venous system to rule out venous reflux disorders. Three cases with bilateral BCRL and three cases with upper limb venous reflux disorder as well as five cases who did not choose combined treatments were ruled out. Ten of the remaining 168 patients lost contact or died of other disease. Finally, 158 patients with BCRL in total were retrospectively included and were followed up one by one.

All the 158 patients included in this study were females, with the age of 57.0 ± 8.3 years. Eighty-six (54.4%) patients had left-sided breast cancer and 72 (45.6) right-sided. All of the 158 patients underwent axillary lymph node dissection and 133 (84.2%) patients received concurrent radiation therapy.

The median (P25, P75) duration of onsets of lymphedema symptoms since breast cancer surgery was 1.0 year, ranged from 0 to 23.0 years during which all patients chose variety of conservative therapies (Table 1). Of all the 158 patients, 142 patients wore compression garments day and night for a period of time, while others chose other conservative therapies. But the results were not satisfactory, which forced them to turn to surgeries. Thirty-four (21.5%) patients had recurrent episodes in 1 year before the visit to hospital and our treatments.

**Table 1:** Conservative therapies before visit to the hospital.

Conservative therapy	Total	Compression garment	Medication	Traditional Chinese medicine	Acupuncture	Cupping	Pressure wave therapy
*N*	158	142	7	4	3	2	1

Before the treatment, the circumferential difference of bilateral arms varied from −3.0 to 23.9 cm, and the volume increase rate varied from 6.56% to 190.4%. All of our 158 patients were at stage II and III of whom 32 (20.3%) suffered 20%–40% volume increase and 120 (75.9%) suffered >40% volume increase. The rest six (3.8%) patients’ arms were swelled unevenly, with the severity of moderate (20%–40% increase of volume) within the forearm or upper arm separately. In view of this, the combination of surgical treatments was chosen in all of these patients with no satisfactory expectation using conservative therapy.

### Response to the combined treatments

**Figure 3:**
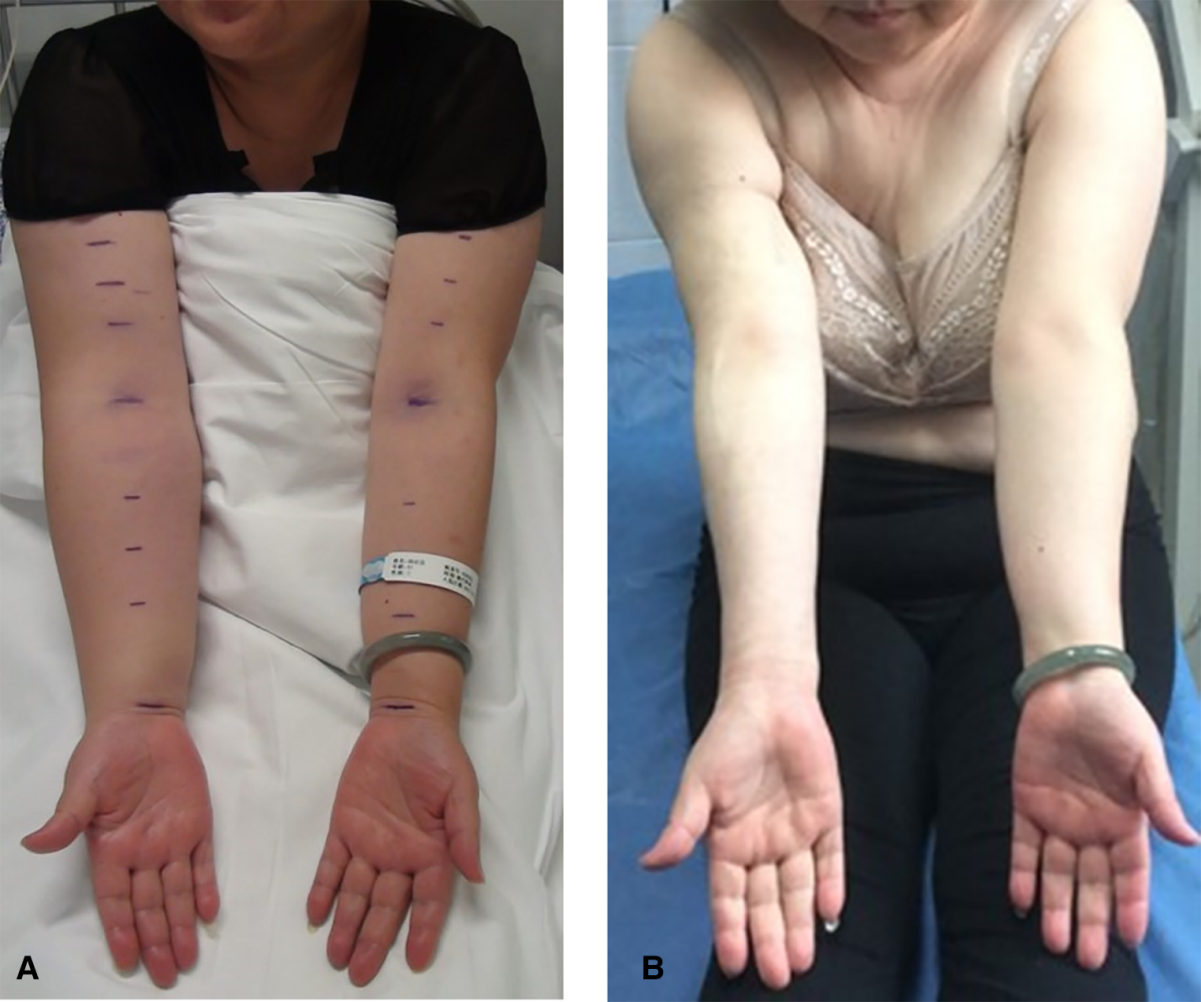
Female, 49 years old, right-sided BCRL: (**A**) before treatments (i.e., combination of liposuction and lymphovenous anastomosis), with the volume increase rate of 45.5%, and (**B**) 32 months after treatments, with the volume increase rate of −11.7%. BCRL, breast cancer-related lymphedema.

All the 158 patients completed the combination treatments of liposuction and LVAs successfully without severe complications. The median (P25, P75) follow-up period was 30 (28, 32) months, ranging from 23 to 36 months [hereinafter referred to shortly as “follow-up(s)”]. The median circumferential difference reduced significantly (*P *< 0.05) from 5.3 cm before treatments to 0.5 cm seven days after treatments, and the median volume difference (volume increase rate) reduced significantly (*P *< 0.05) from 838.3 mL (50.7%) to 7.8 mL (0.6%). The median circumferential difference, volume difference, and volume increase rate kept reducing (*P *< 0.05) surprisingly from 7 days after the surgery to the follow-ups (Figure 3, Tables 2–4).

**Table 2:** Circumferential difference of bilateral arms before and after liposuction followed by lymphovenous anastomosis.

Time point	*N*	Circumferential difference (cm)			Z1/P1	Z2/P2
		Min	Max	M (P25, P75)		
Before treatments	158	0.7	15.9	5.3 (4.1, 6.9)	—	—
7 days after treatments	158	−3.4	6.2	0.5 (−0.8, 1.0)	10.903/0.000^a^	—
Follow-up	158	−2.6	4.9	0.3 (−0.4, 1.0)	−10.903/0.000^a^	−1.982/0.048^a^

M, median; Z1/P1, consequences compared with before treatments; Z2/P2, consequences compared with 7 days after treatments.

^a^ Statistically significant (*P*-value < 0.05).

**Table 3:** Volume difference of bilateral arms before and after liposuction followed by lymphovenous anastomosis.

Time point	*N*	Volume difference (mL)			Z1/P1	Z2/P2
		Min	Max	M (P25, P75)		
Before treatments	158	111.0	3,600.0	838.3 (662.4, 1,129.0)	—	—
7 days after treatments	158	−448.6	1,096.1	7.8 (−120.3, 151.4)	−10.903/0.000^a^	—
Follow-up	158	−344.4	809.7	43.7 (−59.4, 161.1)	−10.903/0.000^a^	−2.173/0.030^a^

M, median; Z1/P1, consequences compared with before treatments; Z2/P2, consequences compared with 7 days after treatments.

^a^ Statistically significant (*P*-value < 0.05).

**Table 4:** Volume increase rate of bilateral arm before and after liposuction followed by lymphovenous anastomosis.

Time point	*N*	Volume increase rate (%)			Z1/P1	Z2/P2
		Min	Max	M (P25, P75)		
Before treatments	158	6.6	190.4	50.7 (40.1, 71.1)	—	—
7 days after treatments	158	−26.3	55.5	0.6 (−7.8, 9.4)	−10.903/0.000^a^	—
Follow-up	158	−20.6	56.60	2.5 (−3.5, 10.2)	−10.903/0.000^a^	−2.108/0.035^a^

M, median; Z1/P1, consequences compared with Before treatments; Z2/P2, consequences compared with 7 days after treatments.

^a^ Statistically significant (*P*-value < 0.05).

The frequency of erysipelas episodes was ranging from 0 to 6 before treatments, while during follow-ups, it was ranging from 0 to 2. Statistically, the frequency of erysipelas episodes also decreased significantly (*P *< 0.05) after the treatments. Originally before our treatments, 33 (20.9%) patients had no more than three episodes every year and 1 (0.6%) patient had six episodes every year. During follow-ups, 134 (84.8%) patients had no erysipelas episodes at all and only 19 (12%) had a one-time episode and 5 (3.2%) twice during all the years after the treatments (Table 5).

**Table 5:** Frequency of erysipelas episodes between before the treatments and during the follow-ups.

Time point	*N*	Min	Max	M (P25, P75)	*Z* value	*P*-value
Before treatments	158	0	6	0.00 (0.00, 0.00)		
Follow-up	158	0	2	0.00 (0.00, 0.00)	−5.011	0.000^a^

M, median.

^a^ Statistically significant (*P*-value < 0.05).

### Dependence on the compression therapy

Compression therapy is the corner stone of conservative therapy as well as a supplementary therapy to help shape the arm after liposuction and help lymph flow after LVAs. Lifelong sustained compression is essential to reduce recurrence. Before the surgeries, 142 patients wore compression garments day and night for a period of time, while the results were not satisfactory. After liposuction followed by LVAs, 10 (6.3%) patients in our study were already independent of compression garments totally during the past 6 months or more. Most patients (144, 91.1%) only wore compression garments during daytime periodically. Only four (2.5%) needed compression all day long. All of the patients’ arms remained stable without recurrence.

## Discussion

Our interest in solving lymphedema started 30 years ago. In the development of lymphedema, thickening and sclerosis of the skin and adipose tissue contains a large amount of fibrotic tissue, which makes the subcutaneous tissue denser than usual (21). The traditional liposuction with negative atmosphere could hardly remove the dense fibrotic tissue. Therefore, we used a PAPLM that can effectively destroy fibrotic and adipose tissue with cannula vibration and shorten the operation time. Additionally, we made the cannulas parallel to the longitudinal axis of the upper limbs during the procedure to reduce damage to lymphatic and blood vessels and prepare for the next LVAs according to Frick’s theory (22).

Lymphatic channels were anastomosed end-to-end or end-to-side to subdermal venules above deep fascia at multiple points traditionally, which was on the traditional anatomic basis that there are no wide connections between the superficial and deep lymphatic vessels, and hypertrophic and proliferative tissue changes in lymphedema occur mainly in the tissue above the deep fascia (23). However, recently, single or multiple deep collecting lymph vessels have been found along neurovascular bundles in the forearm and upper arm, and perforating lymph vessels have been found, which link the superficial and deep lymph vessels (24). All the 158 LVAs procedures were performed using deep lymphatics and brachial vein tributaries beneath deep fascia, which were proved to be effective in improving lymph flow. At the same time, LVAs using deeper lymphatic and blood vessels instead of superficial ones reduced the chance of anastomotic closure from infection or trauma, and LVA procedure beneath the deep fascia would also not be affected by liposuction performed months before. After LVA procedures, lymph fluid flow into veins and lymphatic stasis was ameliorated gradually. Then, the pressure of the lymphatic vessel declined little by little and finally blood reflux occurred at the anastomosis. As a result, thrombus formed and blocked the anastomosis (25, 26). Considering this problem, we improved the LVA procedure by selecting the vein with better valve function to avoid or reduce blood reflux, and reduce the venous pressure at the anastomosis. In terms of the lymphatic vessel, we chose those with a thinner wall with less fibrotic and more obvious contractive function to ensure the flow of lymph fluid. With all these efforts, long-term results after our combined treatments were gratifying.

Based on the above data, we concluded that all the 158 patients’ swelling limbs got relieved after the combined treatments and the condition of the treated arms remained stable from after the treatments to the follow-ups. Therefore, liposuction followed by LVAs for BCRL not only reduced the volume of affected arms and the amount of lymph fluid production but also rebuilt the lymph flow. In this way, the vicious cycle of lymph stasis, fat hyperplasia, and more lymph stasis is broke. After all these systematic surgical treatments and compression therapy, there were patients who already got rid of compression garments. How many of them would be independent of compression therapy at last and return to normal life completely remained to be investigated further.

## Conclusion

Our combined treatments of liposuction followed by lymphovenous anastomosis for breast cancer-related lymphedema received satisfactory results without worrying complications. The volume of affected arms reduced significantly and kept reducing from 7 days after the surgery to at least 22 months later during the follow-ups. After the systematic treatments, some patients got rid of compression garments and returned to normal life again, and there were no recurrences during the follow-ups.

## Data Availability

The raw data supporting the conclusions of this article will be made available by the authors, without undue reservation.
